# The Meeting of the United States Veterinary Medical Association

**Published:** 1892-08

**Authors:** W. L. Hoskins, R. S. Huidekoper

**Affiliations:** Secretary; President


### EDITORIAL.

*To all Members of the United States Veterinary Medical Association and all members of the professsion :*

The Committee of Arrangements has secured a concession of the usual one and one-third rates from the Eastern Passenger Railway Association, which has also been acquiesced in by the Trunk Line and other railway associations. This will be, as usual, a full fare going, with a certificate from the agent, and the one-third fare returning.

I also desire to give notice that all applications for membership must be filed in the Secretary's office on or before September 1st, in order that they may be sure of receiving consideration at the Boston meeting. The afternoon and evening of the second day will be spent by the Association on a trip—the courtesy of the Massachusetts State Veterinary Medical Association.

Two other papers in addition to those announced in the last number of this JOURNAL will be offered for the consideration of the Association.

All State Associations, through their proper officers, will give notice as soon as appointments of delegates are made by their respective associations. The following amendment offered at Washington will come up for action at this meeting:

"Article I. Any applicant for membership shall submit his name upon one of the association's application blanks, duly vouched for by one or more members of the association, or by the resident State Secretary of his respective State. He shall be a graduate of a regularly organized and recognized Veterinary School, which shall have a curriculum of at least three years, of six months each, specially devoted to the study of veterinary science, and whose corps of instructors shall contain at least four veterinarians. If of a medical school a similar curriculum as to time shall prevail."

This alteration to go into effect after the annual meeting of 1892, it shall not be retractive nor apply to applicants who were college matriculants prior to its passage.

W. L. HOSKINS,

*Secretary.*

R. S. HUIDEKOPER,

*President.*

